# Optically secured information retrieval using two authenticated phase-only masks

**DOI:** 10.1038/srep15668

**Published:** 2015-10-23

**Authors:** Xiaogang Wang, Wen Chen, Shengtao Mei, Xudong Chen

**Affiliations:** 1 School of Sciences, Zhejiang A&F University, Linan 311300, China; 2 Department of Electronic and Information Engineering, The Hong Kong Polytechnic University, Hong Kong China; 3 Department of Electrical and Computer Engineering, National University of Singapore, Singapore 117583, Singapore

## Abstract

We propose an algorithm for jointly designing two phase-only masks (POMs) that allow for the encryption and noise-free retrieval of triple images. The images required for optical retrieval are first stored in quick-response (QR) codes for noise-free retrieval and flexible readout. Two sparse POMs are respectively calculated from two different images used as references for authentication based on modified Gerchberg-Saxton algorithm (GSA) and pixel extraction, and are then used as support constraints in a modified double-phase retrieval algorithm (MPRA), together with the above-mentioned QR codes. No visible information about the target images or the reference images can be obtained from each of these authenticated POMs. This approach allows users to authenticate the two POMs used for image reconstruction without visual observation of the reference images. It also allows user to friendly access and readout with mobile devices.

When designing diffractive optical elements that allow for the encryption of data for security applications, phase retrieval algorithms (PRAs) such as the Gerchberg-Saxton algorithm (GSA)^1^, Fienup method^2^ and their derivative^3^ can be used. In 1996, Johnson and Brasher encrypted a biometric image in two phase-only masks (POMs) that together reconstruct an image although neither diffractive element by itself gives any hints as to what is in the image^4^. Under the framework of linear double-random-phase encoding (DRPE) scheme^5^, many modified algorithms in Fourier domain^6^,^7^, Fresnel domain^8^,^9^,^10^,^11^,^12^,^13^,^14^ and gyrator domain^15^ have been proposed to generate POMs for data retrieval. Recently, we have also proposed several methods to produce two POMs for single-image retrieval using iterative nonlinear DRPE^16^,^17^. Degrees of freedom to manipulate the physical parameters of optical waves can be used in those algorithms where an image can be encoded into two or more POMs^4^,^5^,^6^,^7^,^8^,^9^,^10^,^11^,^12^,^13^,^14^,^15^,^16^,^17^. Optical information hiding with POMs has now been one of the most popular application areas for PRAs^18^,^19^,^20^. However, the identifying and the capacity of those computer-generated POMs and the deteriorated original inputs reconstructed optically are still the major concerns in regards to the PRA-based data security protocols.

In this paper, a method for jointly designing two diffractive optical elements having quasi-random phases that allow for the authentication of the elements themselves, the encryption and noise-free retrieval of triple images is proposed based on modified PRAs. No visible information can be obtained from each of the POMs. The target images are inserted into QR codes via hyperlinks that allow for flexible readout with mobile devices. This approach allows users to authenticate the two POMs without visual observation of those images used as references for authentication. The target images can be revealed without visible loss of information due to the property of high damage tolerance capability of QR codes.

## Results

### Optical image reconstruction scheme

**Figure 1:**
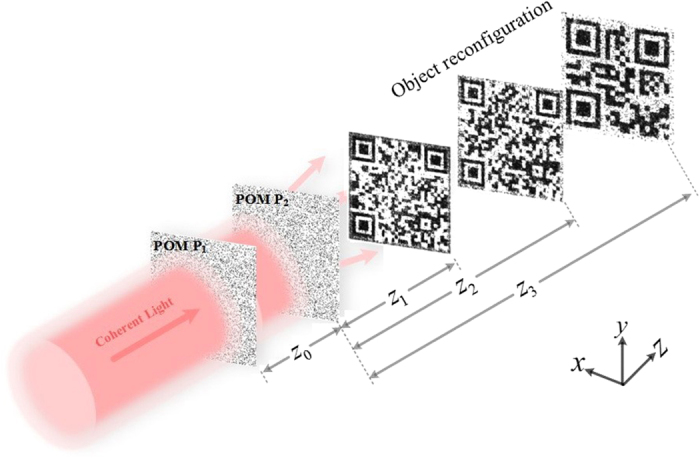
Optical image reconstruction procedure with two authenticated POMs.

The proposed reconstruction procedure of target images is demonstrates in Fig. 1. When the authenticated POM *P*_1_ is illuminated with incident plane wave and then modified by another authenticated POM *P*_2_, the approximates of the input QR codes 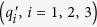 can be detected in three different object planes. Thus, we have


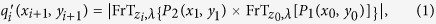


where the operator | | denotes a modulus operation and FrT[•] represents Fresnel transform^17^.

### Image hiding algorithm with sparsity constrains

The procedure of designing two POMs *P*_1_ and *P*_2_ that together reconstruct triple images consists of the following steps: 1. Storing three target images (*f*_1_, *f*_2_, *f*_3_) in QR codes (*q*_1_, *q*_2_, *q*_3_). 2. Generating two sparse POMs (*p*_1*s*_, *p*_2*s*_) from two images (*g*_1_, *g*_2_), which are used as reference for authentication and differ from the target images. 3. Numerical calculation of the two POMs *P*_1_ and *P*_2_ using the sparse POMs and the QR codes.

To generate two sparse POMs, two secret images *g*_1_ and *g*_2_ need to be respectively encoded in two different POMs by using a modified Fresnel domain GSA^11^,^12^ with the two intensity constraints, i.e., a unit amplitude in the input plane and the image to be encoded in the output plane at a certain distance. Note that the two images used as references are not QR codes used for image encoding. The images *g*_1_ and *g*_2_ are independently encoded into POMs *p*_*m*_ and 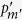 in two iterative processes where the propagation distances are *d*_1_ and *d*_2_. Note that the subscripts *m* and *m*′ represent the number of iterations in iterative processes. When *p*_*m*_ is illuminated with incident plane wave, an approximation of *g*_1_ can be observed in the object plane at distance *d*_1_, which can be written by


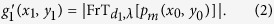


Likewise, we can obtain an approximation of *g*_2_ in the output domain at the distance *d*_2_, which can be given by 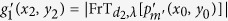.

Sparse representation of the encrypted data can be successfully used for information authentication in some DRPE-based security systems^14^,^21^,^22^,^23^,^24^,^25^. In this step, two sparse phase functions *p*_1*s*_ and *p*_2*s*_ that used for the calculation of *P*_1_ and *P*_2_ can be randomly extracted from the outcomes of the iterative processes, i.e., *p*_*m*_, 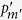. Then the following step is numerical calculation of the two authenticated POMs *P*_1_ and *P*_2_ by using the obtained sparse POMs together with the QR codes. A modified double-phase retrieval algorithm (MDPRA) is proposed to achieve this purpose, where the sparse POMs and the QR codes are used as support constraints. In the MDPRA, let functions 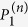 and 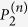 respectively denote the two estimates for *P*_1_ and *P*_2_, where the superscript *n* represents the *n*th iteration of the algorithm. In the initial stage, two random POMs can be used as 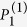 and 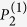, respectively. The QR codes, i.e., *q*_1_, *q*_2_ and *q*_3_, are the three amplitude constraints in the output planes with respect to different distances from the second POM 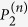, i.e., *z*_1_, *z*_2_ and *z*_3_.

Note that the final solutions for *P*_1_ and *P*_2_ are 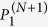 and 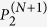 if the iterative stops after *N* iterations. The obtained two POMs *P*_1_ and *P*_2_ require authentication before being applied for optical image retrieval. For brevity, only the identifying of *P*_1_ is explained. The reconstructed signal from *P*_1_ given by 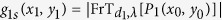 will be compared with the original image *g*_1_, by nonlinear correlation in our proposal, where the two parameters *d*_1_ and *λ* can be used as keys for authentication. The authentication method is described as follows^25^,^26^:


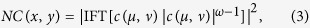


where IFT[•] denotes inverse Fourier transform and *ω* defines the strength of the applied nonlinearity. Function *c*(*μ*, *v*) is given by 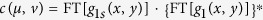, where FT[•] denotes Fourier transform.

### Experimental Simulations and performance analyses

**Figure 2:**
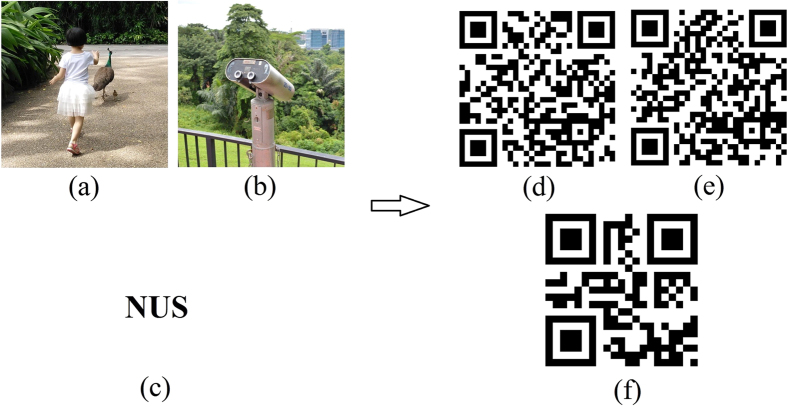
Storing three target images in QR codes. (**a**) Girl, (**b**) Telescope, (**c**) input text information. (**d**–**f**) their respective QR codes. Photographs taken by author.

Figure 2(a,b) are two 500 × 500 color images, which can be respectively inserted into the QR codes shown in Fig. 2(d,e) via hyperlinks. When a user scans the QR code containing the hyperlink which automatically redirects the user to the image. Figure 2(c) shows input the input text information (NUS stands for National University of Singapore) and its respective QR code is presented in Fig. 2(f). All of the QR Codes have the size of 500 × 500. Due to its fast readability, great storage capacity and high damage tolerance capability, storing data in QR codes during the processes of optical encryption^27^,^28^,^29^ and authentication^21^,^22^,^23^ holds many practical advantages. Once the reconstructed QR codes are scanned by smartphones or tablets, the target images can be successfully revealed.

**Figure 3:**
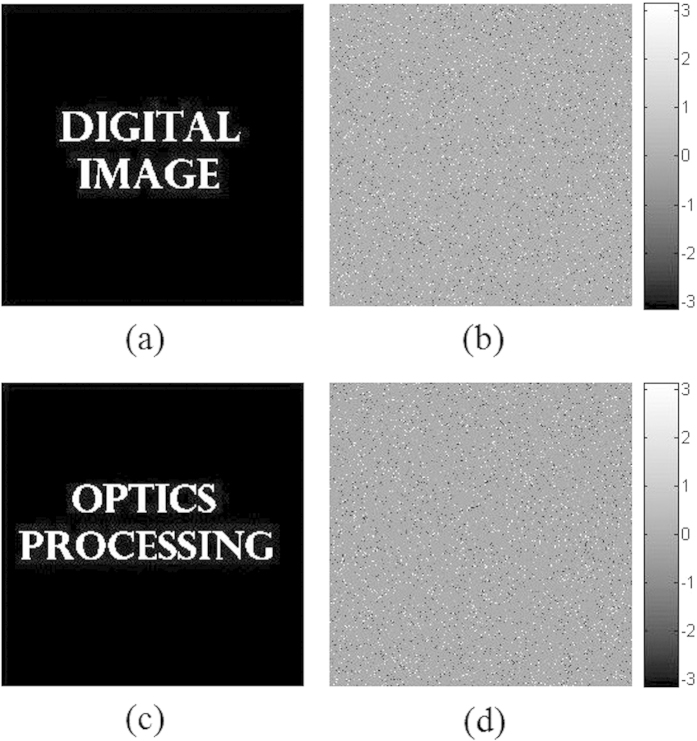
Secret images used as references for authentication and their respective sparse POMs. (**a**) *g*_1_; (**b**) phase distribution of *p*_1*s*_; (**c**) *g*_2_; (**d**) phase distribution of 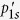.

In our simulations, the pixel dimensions and the illumination wavelength are set as 8*μm* × 8*μm* and *λ* = 633 *nm*, respectively. Figure 3(a,c) show two 500 × 500 pixels binary images used as references for authentication. The propagation distances are given by *d*_1_ = 6 *cm* and *d*_2_ = 8 *cm*, respectively. Figure 3(b,d) demonstrate two sparse phase functions *p*_1*s*_ and *p*_2*s*_ that are randomly extracted from the outcomes of the iterative processes, *p*_50_ and 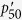, for which both the percentages of the extracted pixels with respect to the pixel size of their originally recovered phase images are 28%.

**Figure 4:**
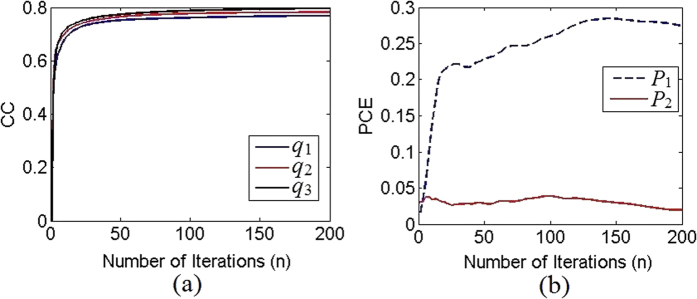
Performance of the proposed iterative algorithm. (**a**) Relation between CC and number of iterations and (**b**) the PCE curves versus the number of iterations.

The two sparse POMs are used as two constraints in the proposed MDPRA, together with the three QR codes. The correlation coefficient (CC) is applied to evaluate the similarity between the recovered images 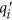 and their original images *q*_*i*_, which is defined by


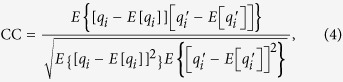


where *E*[] denotes the expected value operator. The CC values get the maximum value of 1 if 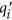 are perfectly correlated with *q*_*i*_. Figure 4(a) shows the relation between number of iterations and CC values (between *q*_*i*_ and 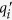), where we set the parameters as *z*_0_ = 8 *cm, z*_1_ = 12 *cm, z*_2_ = 20 *cm* and *z*_3_ = 30 *cm*. It can be seen from Fig. 4(a) that the CC value increases as the number of iterations increases. At the beginning iterations, the three curves shown in Fig. 4(a) overlap almost completely, which implies that the three recovered QR codes have almost identical CC values at the first several iterations. After 100 iterations, the CC values increase very slightly. As expected, two authenticated POMs 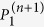 and 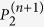 can be generated after the *n*th iteration, which require authentication before being used for image reconstruction. Evaluation of the correlation outputs can be implemented by using peak-to-correlation (PCE)^23^, which is defined as the ratio between the maximum intensity peak value and the total energy of the output plane, usually indicates the sharpness and height of the output correlation peak. Figure 4 shows the two PCE curves obtained by using all correct authentic keys and the nonlinearity strength *ω* = 0.4. Different from the PCE curve corresponding to *P*_2_, the curve with respect to *P*_1_ rises rapidly during the first twenty iterations. After that, the PCE values increased slowly and then reaches a plateau (0.2846) at 145 iteration. However, the PCE values obtained with the second POM *P*_2_ moves up and down in a limited range. It reaches its maximum (0.0390) at 99 iterations. In order to obtain high-quality correlation peak intensity in authentication, the POMs generated after 99 iterations are chosen as the two POMs used for authentication and image retrieval since the CC values increase very slowly after 100 iterations.

**Figure 5:**
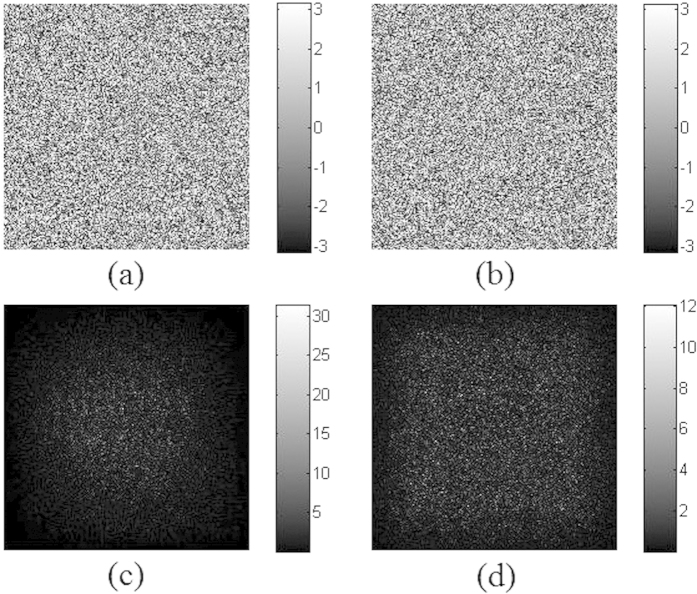
Two obtained POMs and their respective diffractive patterns. Phase distributions (**a**) *P*_1_ and (**b**) *P*_2_, and their respective Fresnel diffraction intensity patterns (**c**) at distance *d*_1_; (**d**) at distance *d*_2_.

The two POMs *P*_1_ and *P*_2_ designed for triple-image reconstruction and obtained after iteration number of 99 are respectively shown in Fig. 5(a,b). They are required for authentication before being used for image reconstruction. The diffraction patterns of *P*_1_ and *P*_2_ are shown in Fig. 5(c,d), at the distances of *d*_1_ and *d*_2_ respectively, from which no information about the two secret binary images can be observed.

**Figure 6:**
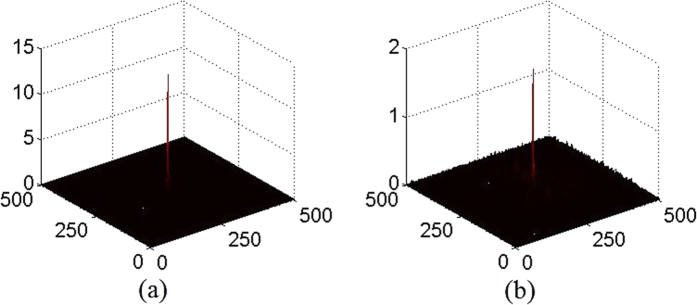
Authentication of the POMs based on nonlinear correlation. (**a**) Correlation outputs corresponding to *P*_1_ and (**b**) *P*_2_.

When those two visually unrecognizable images [Fig. 5(c,d)] are respectively compared with the references, i.e., Fig. 3(a,c), by nonlinear correlation, sharp correlation peaks could be obtained as shown in Fig. 6(a,b), which implies that the two POMs are successfully authenticated with correct parameters.

**Figure 7:**
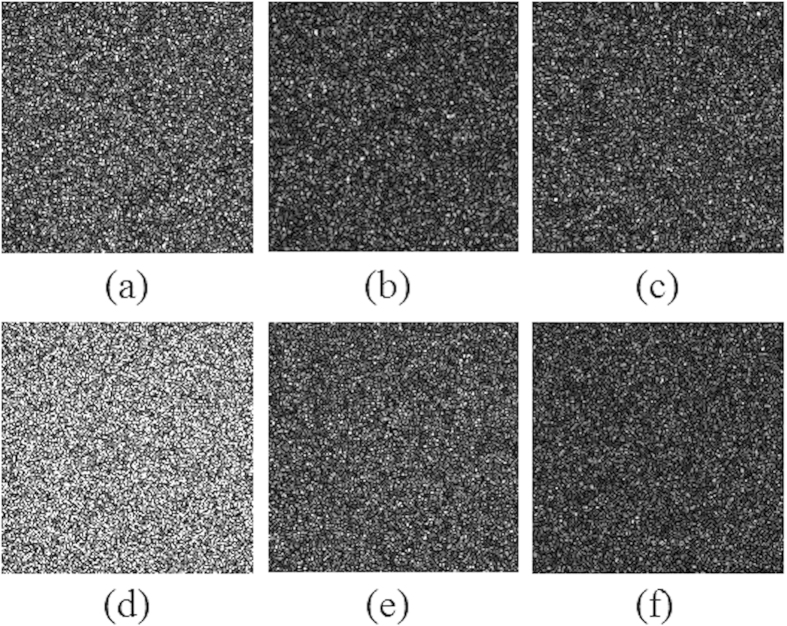
Images reconstructed from only one of the POMs. (**a**–**c**) are the three images reconstructed from *P*_1_ in the different object planes (located from near to far from *P*_1_); (**d**–**f**) are the three images reconstructed from *P*_2_ in the three different object planes.

The security performance of the two POMs is further investigated. When only one of the POMs is placed in the optical scheme shown in Fig. 1, the diffraction patterns in the three object planes are shown in Fig. 7, from which no valuable information about the QR codes could be observed. Note that Fig. 7 only shows the intensity distributions within the area of 4 *mm* × 4 *mm* on the output display. The results shown that neither of the two POMs has the problem of untended information disclosure. The proposed security system can be regard as an information sharing system. Each POM will be sent to different receivers through communication channels. Only when a matched pair of POMs are used for decryption, the primary images can be retrieved by scanning the decrypted QR codes.

**Figure 8:**
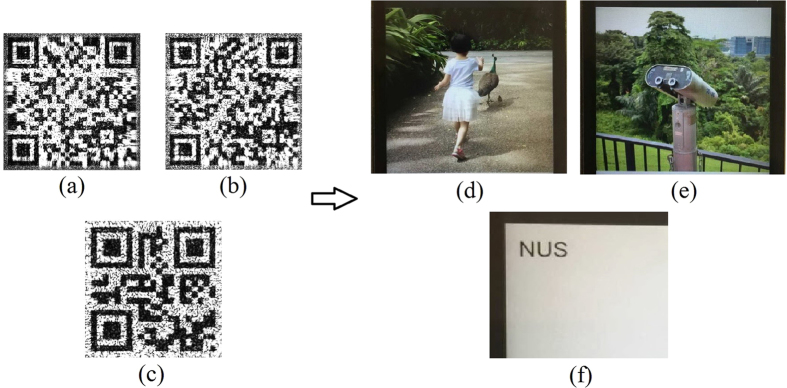
The reconstructed QR codes from *P*_1_ and *P*_2_ at different object planes and their respective retrieved images using a smartphone.

Results for the reconstructed images from the two POMs *P*_1_ and *P*_2_ in different object planes using all the correct physical and geometric parameters are shown in Fig. 8(a–c), which have worse quality than the original images due to the effect of energy loss. The energy of the above three recovered images respectively account for about 85%, 84% and 79% of the total energy in their corresponding object planes. Smartphone was used to display the decrypted images by scanning the reconstructed QR codes. As demonstrated in Fig. 8(d–f), the two target color images and the text information can be retrieved without visible loss of information.

## Discussions

We developed an algorithm for jointly designing two POMs that allow for the encryption and noise-free retrieval of triple images. Compared with previous works, the proposed algorithm based on sparsity constraints and QR codes has the following features:

- This approach allows users to authenticate the two POMs without visual observation of those images used as references for authentication. Since a huge number of differently encoded POMs can be sent out through communication channels^14^, the identifying and matching of the POMs in a simple and direct manner can help increase efficiency. The MPRA with sparsity constraints may expect to be used in the computation of digital holograms and meta-holograms^30^,^31^,^32^ for image display and information authentication.
- There is no problem of information disclosure in the proposed method. No information can be visually observed from the two POMs and their respective diffraction patterns. Our method can also be used for encryption of three-dimensional objects.
- It allows user to friendly access and readout with mobile devices. The target images can be revealed without visible loss of information due to the property of high damage tolerance capability of QR codes. Optically secured information retrieval can be realized when the two designed POMs manufactured by a number of techniques including embossing on plastic films and encoding on photopolymer are placed in the proposed scheme shown in Fig. 1.

## Methods

### Double-phase retrieval algorithm with sparsity constraints

Let functions 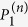 and 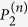 respectively denote the two solutions for *P*_1_ and *P*_2_, where the superscript *n* represents the *n*th iteration of the algorithm. In the initial stage, two random POMs can be used as 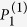 and 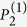, respectively. The QR codes, i.e., *q*_1_, *q*_2_ and *q*_3_, are the three amplitude constraints in the output planes with respect to different distances from the second POM 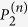, i.e., *z*_1_, *z*_2_ and *z*_3_. The process proceeds as follows:

(i) The first POM 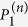 illuminated with incident plane wave is first Fresnel-transformed at the propagation distance *z*_0_. The resultant wave function can be written as


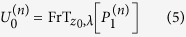


which is then multiplied by the second POM 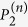 and propagates forward to the output plane through distances *z*_*i*_ (*i* = 1, 2, 3) to obtain new wave functions


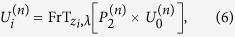


where the coordinates are omitted for simplicity.

(ii) Replace amplitude parts of the diffraction space wave functions 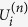 with amplitude constraints *q*_*i*_. Then the modified functions in the three output planes simultaneously transform back to the plane where the second POM 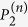 locates to get a new wave function 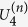.


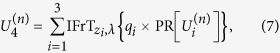


where IFrT[•] represent inverse Fresnel transform and PR[•] denotes phase reservation, retaining the phase of a complex function but truncating its amplitude part.

(iii) Update the two input POMs 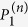 and 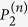 with 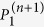 and 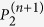. First, we obtain a POM function 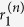 and then update the input POM 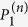 with 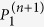 using 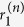, which can be written as


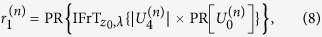



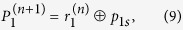


where the symbol ⊕ denotes a particular way of data embedding. For clarity, the calculation of 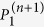 described by Eq. (9) is explained. We first extract the non-zero pixels of POM *p*_1*s*_ as the data background for POM 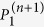, and then embed the pixel values of 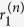 into 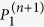 pixel by pixel but keep the background unchanged.

To generate another POM used for next iteration, we first compute a POM function 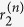 by using 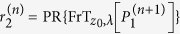 and then obtain POM 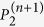 by


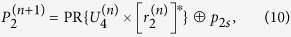


where the superscript * denotes conjugation.

(iv) Repeat steps (i)–(iii) until the preset threshold value is satisfied. Suppose the iteration process stop at the *N*th iteration. The convergence of the algorithm is demonstrated by the relation between the CC values [between *q*_*i*_ and their approximates 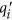 obtained by substituting the two POMs computed with Eqs. (9) and (10) into Eq. (1)] and the iteration numbers.

**Figure 9:**
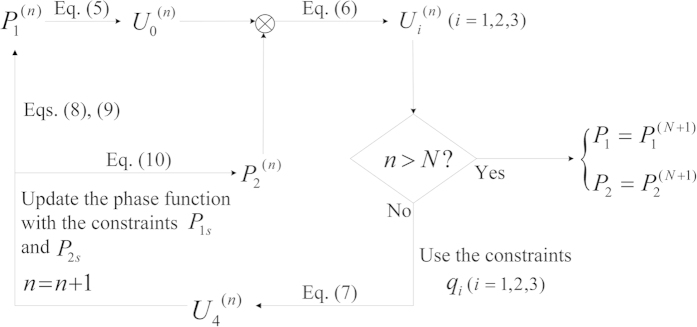
Flowchart of the proposed double-phase retrieval algorithm.

To sum up, a flowchart of the iterative phase retrieval algorithm is depicted in Fig. 9. So far, we have obtained the two POMs *P*_1_ and *P*_2_, which require authentication before being applied for optical image retrieval and can be respectively represented by function 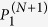 and 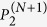. In general, the amplitude parts of function 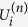 would be closer to the constraints *q*_*i*_ with the increasing of iterations until stagnation. After *N* iterations, the estimates of *q*_*i*_ are denoted by 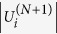. From Eqs. (1), (5) and (6), we can readily obtain 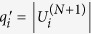, which implies that the finally recovered images 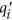 could be expected to be closer to their original images *q*_*i*_ by increasing the number of iterations before reaching plateau values. It should be pointed out that the MDPRA is designed under the framework of nonlinear double random phase encoding. Different from previously proposed methods of single image encoding^16^,^17^, here we encode three QR codes into two POMs in Fresnel domain. Two sparse POMs calculated from two reference images for authentication are used as constraints in the iteration process.

## Additional Information

**How to cite this article**: Wang, X. *et al.* Optically secured information retrieval using two authenticated phase-only masks. *Sci. Rep.* **5**, 15668; doi: 10.1038/srep15668 (2015).
